# The Burden of Retinopathy of Prematurity in Albania: A Nationwide Observational Study

**DOI:** 10.7759/cureus.97355

**Published:** 2025-11-20

**Authors:** Eglantina Bulica, Vilma Mema, Alketa Tandili, Ilir Arapi, Spiro Dama, Amarilda Kanani

**Affiliations:** 1 Ophthalmology, University of Medicine, Tirana (UMT), Tirana, ALB; 2 Pediatric Intensive Care Unit, University of Medicine, Tirana (UMT), Tirana, ALB; 3 Public Health, University of Medicine, Tirana (UMT), Tirana, ALB

**Keywords:** albania, birth weight, gestational age, preterm infants, retinopathy of prematurity, screening outcomes

## Abstract

Background: Retinopathy of prematurity (ROP) is a vasoproliferative disorder of the developing retina and a leading cause of preventable childhood blindness. While data from high- and middle-income countries are well-documented, Albania has until now lacked national-level information on ROP incidence and severity.

Objective: This study aimed to report the first national data on ROP screening outcomes in Albania and to assess the prevalence, severity, and clinical characteristics of ROP in preterm infants. The study also explores perinatal risk factors associated with the severity of ROP.

Methods: A prospective observational study was conducted across neonatal intensive care units (NICUs) in Albania. A total of 173 preterm infants who met the international screening criteria (≤31 weeks gestational age (GA) or ≤1500 g birthweight (BW)) or with comorbidities underwent indirect ophthalmoscopy. Data regarding GA, BW, ROP stage, plus disease, and treatment requirements were collected and analyzed.

Results: Of the 173 infants screened, 88 (50.9%) had no ROP, 65 (37.6%) developed ROP of any stage, and 18 (10.4%) had incomplete data. Among the 65 infants with ROP, 45 (26.0%) had stages 1-2, 12 (6.9%) had stage ≥3, and 3 (1.7%) presented with plus disease. Infants with advanced ROP had a mean GA of 26.9 ± 1.3 weeks and a mean BW of 1313 ± 217 g. Severe ROP was significantly associated with both lower GA (p < 0.001) and BW (p < 0.001). Two infants with severe ROP required mechanical ventilation.

Conclusion: This first national study demonstrates that more than one-third of preterm infants in Albania develop ROP, with nearly 7% progressing to advanced stages. The findings highlight the urgent need for a structured national screening program, improved neonatal care, and access to treatment.

## Introduction

Retinopathy of prematurity (ROP) is a potentially blinding retinal disorder affecting premature infants, particularly those of low birth weight (BW) and early gestational age (GA) [1]. Advances in neonatal care have improved survival rates globally, yet the incidence of ROP has increased in many low- and middle-income countries where systematic screening programs may be absent [2,3].

Albania, a transitioning healthcare system, has until now lacked published national data on ROP. The absence of such data impedes effective prevention strategies and policy development. This study represents the first nationwide effort to assess the incidence, severity, and associated risk factors for ROP in Albanian neonatal intensive care units (NICUs).

## Materials and methods

Study design

This was a prospective observational study carried out in two NICUs in Albania. The aim was to evaluate the incidence and characteristics of ROP and to identify major perinatal factors associated with its development. Data collection was prospective and based on routine clinical screening examinations.

Study population

The study included preterm infants admitted to NICUs between September 2023 and September 2024 who met the established international screening criteria for ROP: GA ≤ 32 weeks and/or BW ≤ 1500 g, as well as selected larger infants with an unstable clinical course requiring prolonged oxygen therapy.

According to the General Directorate of Civil Status, there were 7,183 live births in Tirana during 2023, of which approximately 8%-10% (around 610) were premature. Applying the screening criteria, an estimated 28.3% (173 infants) required ophthalmologic examination and were included in the study.

Infants with incomplete medical records or poor-quality retinal documentation were excluded.

Sample size

A total of 173 preterm infants met the inclusion criteria and were examined for ROP. This number represents all eligible infants available for complete assessment during the study period.

Study measures

Information was collected through a structured form covering demographic, perinatal, and ophthalmologic data.

Perinatal data included GA, BW, sex, mode of delivery, need and duration of mechanical ventilation, oxygen exposure, blood transfusions, and associated neonatal complications.

Ophthalmologic data included the stage and zone of ROP, presence of plus disease, and need for treatment, classified according to the International Classification of Retinopathy of Prematurity (ICROP).

Screening examinations were performed by ophthalmologists using indirect ophthalmoscopy after pupil dilation with 1% tropicamide and 2.5% phenylephrine. Follow-up intervals were determined by retinal findings.

The first examination was performed at 4-6 weeks postnatal age, followed by serial examinations every 1-2 weeks (depending on the stage of retinal vascularization development) until full vascularization or regression of disease. 

Ethics statement

The study followed the ethical principles outlined in the Declaration of Helsinki. Ethical approval was granted by the Institutional Review Board of the University Hospital Center “Mother Teresa,” Tirana. Written informed consent was obtained from the parents or legal guardians of all participating infants.

Statistical analysis

All data were entered and analyzed using IBM SPSS Statistics for Windows, Version 27.0 (Released 2019; IBM Corp., Armonk, NY, USA). Descriptive statistics were calculated for all study variables. Continuous variables were expressed as mean ± standard deviation (SD), and categorical variables as frequency and percentage.

Comparisons between infants with and without ROP were made using the chi-square test for categorical variables and the independent samples t-test for continuous variables. A p-value < 0.05 was considered statistically significant.

## Results

Screening outcomes

**Figure 1:**
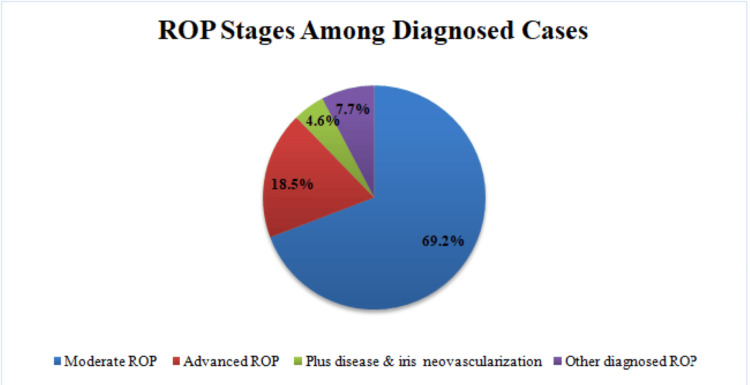
Screening outcomes of preterm infants in Albania

Among the 173 infants screened, 88 (50.9%) had no ROP, 65 (37.6%) had an ROP of any stage, and 18 (10.4%) had incomplete or missing follow-up data (Figure 1).

**Figure 2:**
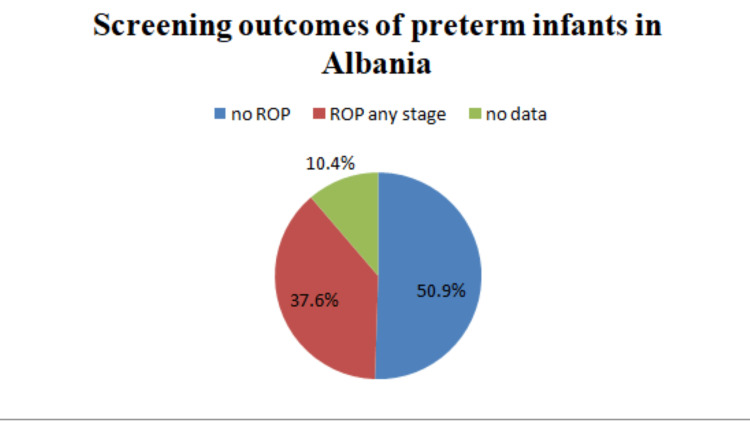
ROP stages among diagnosed cases

Among the 65 cases diagnosed with ROP, 45 (69.2%) had moderate ROP requiring close monitoring, 12 (18.5%) were in the advanced stage, 3 (4.6%) presented with plus disease and iris neovascularization requiring immediate treatment, and 5 (7.7%) were classified as other diagnosed ROP cases (Figure 2).

ROP stage distribution

**Figure 3:**
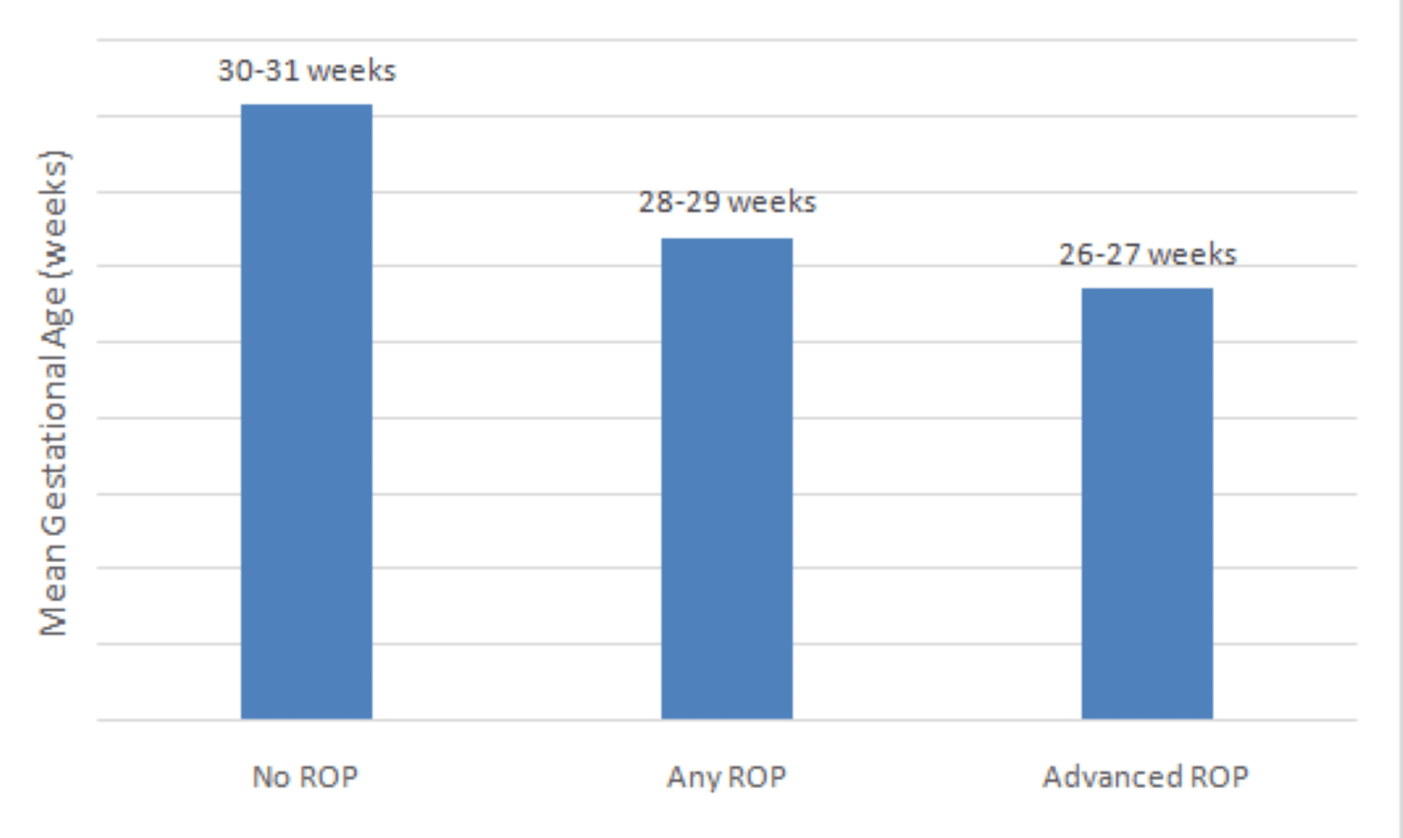
Distribution of ROP stages among the affected infants

Of the 65 infants with ROP, 45 (26.0%) were classified as stages 1-2, 12 (6.9%) as stage ≥3, and 3 (1.7%) as plus disease (Figure 3).

Gestational age

**Figure 4:**
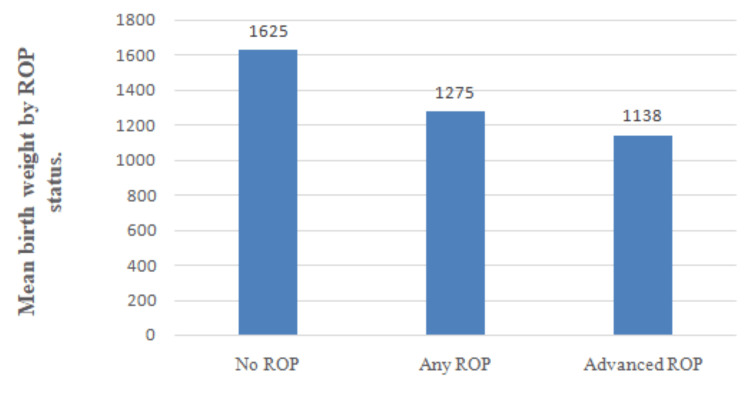
Mean gestational age by ROP status ROP: retinopathy of prematurity.

The mean GA of infants without ROP was 31.1 ± 2.2 weeks, those with any ROP was 28.4 ± 1.9 weeks, and those with advanced ROP was 26.9 ± 1.3 weeks. Quantitative results are summarized in Table 1, and their graphical representation is provided in Figure 4. Lower GA was significantly associated with higher ROP severity (p < 0.001).

**Table 1:** Clinical characteristics by ROP status ROP: retinopathy of prematurity.

Group	Mean GA (weeks) ± SD	Mean BW (g) ± SD	% of group with ROP
No ROP (n = 88)	31.1 ± 2.2	1625 ± 310	0%
Any ROP (n = 65)	28.4 ± 1.9	1275 ± 295	100%
Advanced ROP (n = 12)	26.9 ± 1.3	1138 ± 222	100%

Birth weight

**Figure 5:**
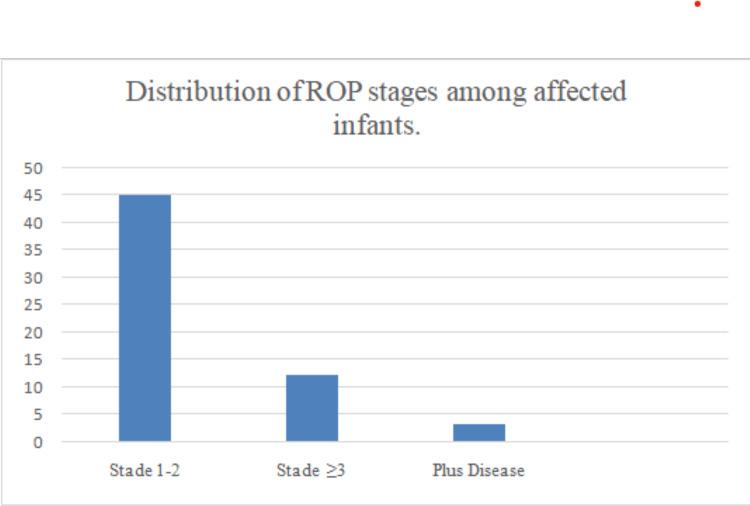
Mean birth weight by ROP status ROP: retinopathy of prematurity.

The mean BW of all screened infants was 1480 ± 385 g. The mean BW of infants without ROP was 1625 ± 310 g, those with any ROP was 1275 ± 295 g, and those with advanced ROP was 1138 ± 222 g. Quantitative results are summarized in Table 1, and their graphical representation is provided in Figure 5. Lower BW was significantly correlated with ROP incidence (p < 0.001).

Respiratory support

Among the 12 infants with advanced ROP, 2 required mechanical ventilation. Interestingly, two infants developed severe disease without ventilation, suggesting a multifactorial etiology beyond oxygen therapy.

## Discussion

This first national report provides valuable insight into the epidemiology of ROP in Albania, revealing that 37.6% (n = 65) of screened preterm infants developed some degree of ROP, while 6.9% (n = 12) progressed to advanced stages requiring consideration of treatment. Because ROP progresses sequentially, timely and periodic screening of at-risk preterm infants is essential to identify retinal changes before they become vision-threatening [4]. The incidence observed is markedly higher than that reported in most Western European countries, where improvements in neonatal care and systematic screening protocols have reduced the prevalence to approximately 15%-25% [5,6]. However, the Albanian data are comparable to those described in other middle-income or transitioning healthcare systems, such as in Eastern Europe, Latin America, and parts of Asia [2,7,8].

GA and BW were identified as the most important predictors of disease development, consistent with well-established international findings [1,3,9]. The mean GA of 26.9 weeks and BW of 1313 g among infants with advanced ROP closely parallels international treatment thresholds defined by the Early Treatment for Retinopathy of Prematurity (ETROP) study [10]. Importantly, ROP was not limited to extremely low-BW infants, as several infants with BW > 1200 g also developed clinically significant disease. This suggests that the Albanian neonatal population may face additional risk factors, such as inconsistent oxygen monitoring, fluctuating nutritional support, and limited access to advanced neonatal care technologies, which can increase susceptibility even among relatively heavier preterm infants [11].

Another important finding is the occurrence of severe ROP in infants who had not received mechanical ventilation. While mechanical ventilation and supplemental oxygen are well-recognized contributors to ROP pathogenesis, the absence of these interventions in some severe cases underscores the multifactorial nature of the disease. Sepsis, inadequate weight gain, blood transfusions, and nutritional deficiencies are among the co-factors associated with heightened risk [12].

Equally noteworthy is the 10% rate (n = 18) of incomplete data and loss to follow-up. This reflects systemic challenges in continuity of care, communication between NICUs and ophthalmology teams, and the absence of integrated medical record systems. Experiences from other countries demonstrate that centralized electronic registries and mandatory screening protocols can substantially reduce follow-up losses [13].

Overall, the findings indicate that Albania is experiencing what has been termed the “third epidemic” of ROP, characterized by increased survival of preterm infants without parallel development of screening and treatment infrastructure [1]. Without timely intervention, this epidemic may lead to an increased burden of preventable childhood blindness in the coming years. There are some limitations regarding the data because 18 infants (10.4%) were lost to follow-up or had incomplete medical records, and 2 eyes could not be assessed due to inadequate examination of the babies.

## Conclusions

This national study establishes, for the first time, that more than one-third of Albanian preterm infants develop ROP, with nearly 8.6% (n = 15) progressing to advanced stages associated with a high risk of visual impairment. These findings clearly demonstrate that ROP is an emerging public health problem in Albania. To address this burden, several urgent steps are needed. First, developing and implementing a national ROP screening protocol is essential to ensure the timely identification of at-risk infants. Such protocols should align with international guidelines while being adapted to Albania’s specific demographic and healthcare context. Second, neonatal and ophthalmic capacity must be expanded. This includes training NICU staff in appropriate oxygen management, equipping units with reliable monitoring systems, and ensuring that ophthalmologists are adequately trained and resourced to provide both screening and treatment (laser therapy and anti-VEGF injections). Third, integrating standardized data collection systems is critical. Establishing a national electronic ROP registry would support long-term surveillance, improve follow-up, and provide the evidence base needed to guide healthcare policy.

More broadly, the findings underscore the need for investment in neonatal care quality and stronger interdisciplinary collaboration between neonatologists and ophthalmologists. Without coordinated action, Albania risks a growing number of children with preventable blindness, imposing significant lifelong social and economic burdens on families and society. Conversely, the timely implementation of screening and treatment programs offers an opportunity to dramatically reduce visual impairment and improve outcomes for vulnerable preterm infants; an estimated 15 children each year are at risk of blindness without timely diagnosis. By adopting these measures, Albania can move from documenting ROP as an emerging clinical concern to actively preventing blindness in its most vulnerable infants, joining the ranks of countries that have successfully reduced the burden of ROP through coordinated national efforts.
